# Effect of dry needling, ischemic compression and cross-taping of the masseter in patients with orofacial myofascial pain: a randomized comparative study

**DOI:** 10.3389/froh.2024.1524496

**Published:** 2025-01-07

**Authors:** B. Macedo de Sousa, N. López-Valverde, A. López-Valverde, D. Neves, M. Santos, J. A. Blanco Rueda

**Affiliations:** ^1^ Institute for Occlusion and Orofacial Pain, Faculty of Medicine, University of Coimbra, Coimbra, Portugal; ^2^ Department of Surgery, Faculty of Medicine, University of Salamanca, Salamanca, Spain; ^3^ Biomedical Research Intitute of Salamanca (IBSAL), Salamanca, Spain

**Keywords:** orofacial myofascial pain, dry needling, ischemic compression, cross-taping, masseter

## Abstract

**Background and objective:**

Temporomandibular disorders, of multifactorial etiology, refer to a series of pathologies that affect the temporomandibular joint and the associated musculature of the orofacial region and are the result of alterations in the physiological relationships of the stomatognathic system, responsible for functions such as chewing, phonation and swallowing. They produce, among other symptoms, mainly pain, which affects the quality of life of the patients who suffer from them. To alleviate the discomfort of neuromuscular pathology in the orofacial region, various therapeutic strategies are employed, ranging from non-invasive to more invasive methods. The aim of the study was to compare the efficacy of three therapeutic methods (dry needling, ischemic compression and cross-taping) in reducing or relieving masseter pain in individuals with orofacial myofascial pain.

**Materials and methods:**

A multicenter randomized comparative clinical trial was conducted in 60 subjects over 18 years of age, divided into three groups: dry needling, ischemic compression and cross-taping. Pain intensity was assessed, randomly, by a single blinded evaluator, according to the Numerical Pain Rating Scale in the pre-treatment period, immediately after, 1–2 weeks and one month later.

**Results:**

Immediately after applying the therapies, there was a greater decrease in pain intensity in dry needling, followed by ischemic compression and a smaller decrease in the cross-taping technique (*p* < 0.0001; *p* = 0.0001; *p* = 0.0014, respectively). After 1–2 weeks, there was a noticeable increase in the dry needling technique, however, there was a decrease in pain in the cross-taping technique. After 1 month of application, both dry needling and ischemic compression showed a slight reduction in pain intensity, in contrast to the cross-taping group, which showed an increase in pain intensity.

**Conclusions:**

Dry needling and ischemic compression were more effective than cross-taping for immediate reduction of orofacial myofascial pain. Further short- and long-term research is needed to confirm these findings.

**Clinical Trial Registration:**

clinicaltrials.gov, identifier (NCT0660604).

## 1 Introduction

Temporomandibular disorder (TMDs) refers to a spectrum of clinical conditions that impact the temporomandibular joint (TMJ) and associated musculature within the orofacial region. These disorders result from disruptions in the physiological relationships within the Stomatognathic System (SS), which is responsible for functions such as mastication, speech, and swallowing (1, 2). TMDs are prevalent and are estimated to affect 60%–70% of the population (3), with a higher incidence observed in young adult women (4), although they also commonly affect older people, especially in the context of degenerative joint diseases such as osteoarthritis (5). The clinical presentation is typically characterized by pain, fatigue, joint noise and irregular or limited jaw function, which has a significant impact on quality of life (6, 7). Treatment is challenging and focuses on pain control, reduction of excessive load, restoration of muscle function and functional normality of the TMJ (8, 9). The etiology of TMDs is now understood to be multifactorial (6, 10), involving predisposing, precipitating and perpetuating factors such as gender, trauma, psychosocial conditions (e.g., anxiety, depression), hormonal influences, anatomical variations and oral parafunctions (2, 8). Although traditional gnathological principles suggested a causal relationship between occlusal factors and TMDs, current evidence does not support this association (11), as SS structures can physiologically adapt to changes in occlusion. This shift in understanding marks a significant departure from the early theories of TMDs proposed by Costen (12, 13).

The Diagnostic Criteria (DC) for TMDs (DC/TMDs) is the main diagnostic tool recommended by the world's leading scientific societies for the evaluation of these disorders (14). It uses scientifically validated methods to facilitate clinical examination and the collection of information on the clinical signs of TMDs. In addition, the CD/TMDs assesses behavioral, psychological and psychosocial factors associated with the individual, providing a comprehensive and reliable diagnostic framework. The system is divided into two axes: Axis I focuses on physical and clinical examination to establish a probable diagnosis, while Axis II addresses psychosocial aspects, assessing pain severity, disability and the role of emotional factors (15). Although the CD/TMD is the most widely used and reliable protocol, it has limitations, such as not covering all pathologies, including hypertrophy and congenital anomalies, for example (16). Muscle palpation is a vital diagnostic tool for identifying TMDs and should take into account the DC/TMDs guidelines. There are two palpation techniques: pressure palpation, used for muscles with underlying bone, and pincer palpation, suitable for muscles such as the sternocleidomastoid and upper trapezius, which lack such support (17). Muscles should be palpated in a relaxed state, following the orientation of their fibers. Palpation should cover the origin, body and insertion of the muscle, applying a pressure of approximately 1 kg and holding it for 5–10 s to accurately detect pain propagation patterns (18). The masseter muscle, the main muscle involved in mastication, plays a crucial role in the elevation of the mandible against the maxilla to generate masticatory force. Anatomically, it is characterized by its quadrilateral shape, with well differentiated superficial and deep layers. For palpation of this muscle and to gather an accurate assessment, pressure should be applied evenly against the underlying mandibular bone and perpendicular to the orientation of the fibers, due to their vertical arrangement (19).

Band tightness is a common muscle disorder characterized by myofascial trigger points, which are small hyperirritable nodules within muscle fibers (20). These nodules, when palpated, reveal hypersensitive and abnormally stiffened muscle fibers, indicating increased tension (21) and may cause localized pain, pain referred to distant areas, or a twitch response (22, 23). They may also trigger autonomic reactions, such as muscle weakness, paresthesia, pruritus, pallor, sweating, rhinorrhea, ptosis and nausea (24–26).

There are two types of myofascial trigger points: latent and active. Latent trigger points cause symptomatology by provocation and pain by palpation. In contrast, active trigger points cause spontaneous pain both at the origin and at the referral sites, with symptoms that are clinically evident (27). The status of these trigger points can fluctuate depending on factors such as psychological stress and muscle overload (28, 29).

According to the Integrated Hypothesis, proposed by Simons & Travell in 1996, myofascial trigger point formation is due to excessive release of acetylcholine at the motor endplate, which causes localized contraction of muscle fibers and increased tension, leading to hypoxia and accumulation of sensitizing substances (30). This results in hyperalgesia and reduced levels of acetylcholinesterase, which perpetuates the tenderness and the existence of the trigger point even after the initial cause has resolved (31).

Various therapeutic strategies are employed to alleviate the pain and discomfort of neuromuscular pathology in the orofacial region, ranging from non-invasive to more invasive methods (32). The consensus is to start with less invasive treatments, reserving more aggressive approaches for cases in which symptoms significantly impact the patient's quality of life, or when initial treatments fail (33). A promising therapy for muscle involvement is dry needling, which involves inserting a sterile fine monofilament needle into hyperirritable muscle nodules without injecting or removing substances (34, 35). This technique can be superficial (Baldry's technique), in which the needle penetrates up to 10 mm into the subcutaneous tissue, or deep, aimed directly at the myofascial trigger point (36). The aim is to induce controlled muscle microspasms and their subsequent relaxation, it being important that the needle does not remain in place for long periods of time, in order to avoid complications (37).

Ischemic compression is another noninvasive treatment option for myofascial trigger points. This manual technique involves applying pressure for 30 to 90 s to normalize the biomechanical properties of muscle fibers, reduce pain and restore muscle function (38, 39). The procedure temporarily restricts local blood flow (causing local ischemia), followed by a surge of oxygenated blood on release, which improves muscle metabolism and thus healing (40).

A third therapeutic option, increasingly popular in Europe, is the use of cross-taping (41). These tapes consist of three or four polyester strips arranged in an equidistant cross shape and coated with a non-elastic adhesive. They are applied over myofascial trigger points at a 45° angle to the muscle fibers. The tapes provide muscle support, improve fiber stability, relieve pain, aid lymphatic drainage, and improve blood circulation (42). For effective application, the skin should be cleaned with alcohol and dried, and they should not remain in place for more than 24 h to avoid displacement or irritation (43).

To date, there is a lack of research on the use of cross-taping in the treatment of myogenic TMD, as well as on the comparison between this therapy, dry needling and ischemic compression. Therefore, the aim of this study was to compare the efficacy of these three therapeutic methods in reducing or relieving masseter pain in individuals with orofacial myofascial pain.

For this reason, we considered it of interest to carry out a comparative study between the three techniques described above. The efficacy of each technique was evaluated by periodic follow-up of the patients for one month.

## 2 Methods

### 2.1 Study design; registration

A multicenter randomized compared clinical trial (RCT) was designed according to CONSORT guidelines at the University Hospital of the Faculty of Medicine of the University of Coimbra (Portugal) and at the Dental Clinic of the University of Salamanca (Spain). The research was conducted in accordance with the 1975 Declaration of Helsinki, revised in 2000 and approved by the Ethics Committee of the Faculty of Medicine of the University of Coimbra (protocol number CE-092/2023). The study was registered in ClinicalTrials.gov: ID NCT06606041. All patients included were given the corresponding informed consent, which they approved before the start of the study.

### 2.2 Patients; inclusion and exclusion criteria

A sample of 60 patients was recruited, which we considered a reasonable number. Inclusion criteria were patients with myofascial pain diagnosed according to the diagnostic criteria for DC/TMD and over 18 years of age. Exclusion criteria were pregnant women; children under 18 years of age; other pathologies within the spectrum of DC/TMDs; anticoagulated patients and patients with antiplatelet treatments; diabetes mellitus; fibromyalgia; hematologic pathologies; acute phase autoimmune pathologies; neurologic pathologies and malignant tumor pathologies; patients with aicmophobia and patients under chronic medication that interferes with pain, such as opioids, antidepressants and muscle relaxants. Patients were randomly assigned (RADOMIZE.NET®, Ottawa, Ontario, Canada) to each of the three groups, taking into account the three different treatment methods used in this study: dry needling, ischemic compression and the application of cross tapes.

### 2.3 Treatments

**Figure 1:**
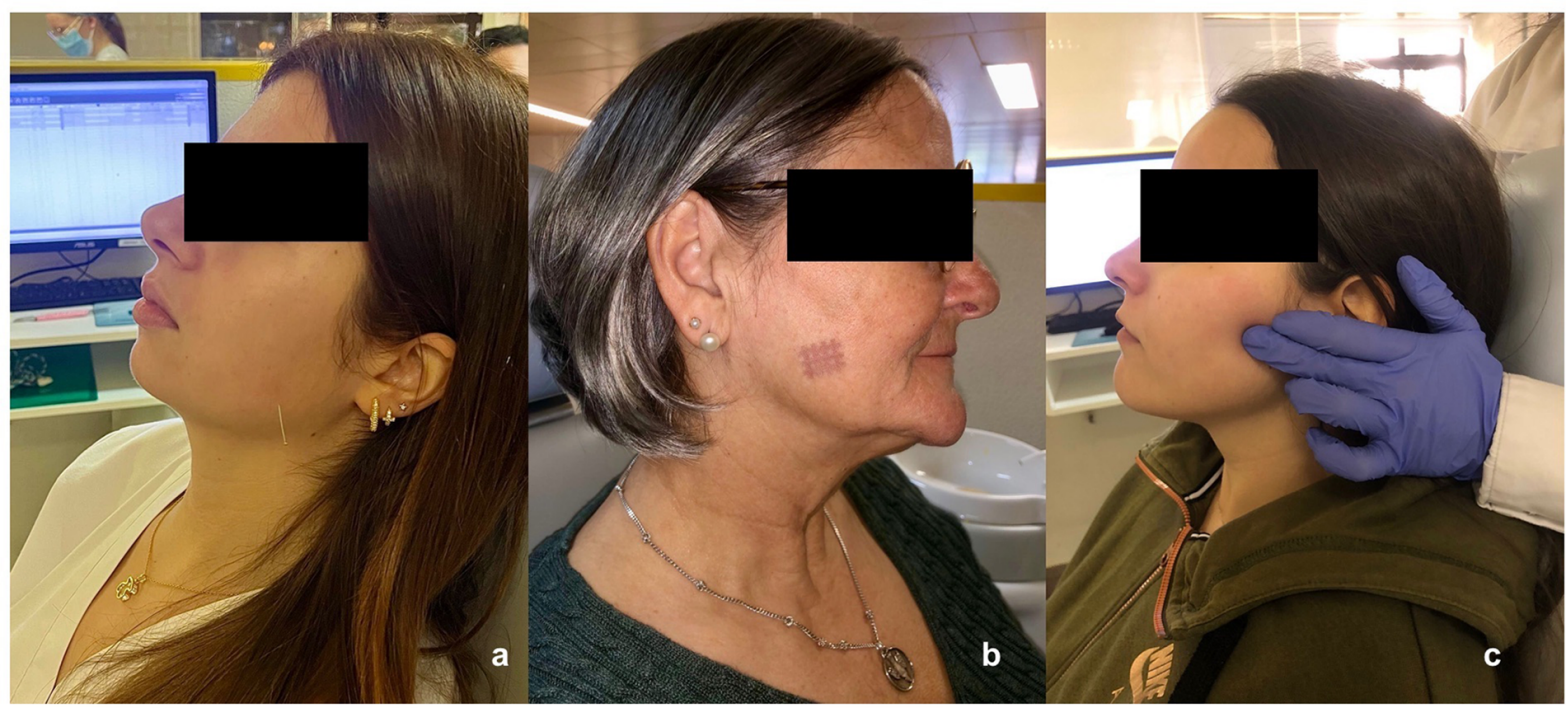
**(a)** Dry needling; **(b)** cross-taping and **(c)**, ischemic compression treatments.

The following protocol was followed: For the dry needling procedure, the trigger point area was first sterilized with a compress soaked in alcohol. Then, a 0.25 × 13 mm needle was carefully introduced into the taut band, using small controlled back-and-forth movements until a local contraction response was obtained. After removing the needle, the area was cleaned with a compress to treat any slight local bleeding that may have occurred (Figure 1A). As for the cross-taping group, the trigger point area was also disinfected with an alcohol-soaked compress and then dried. The cross tape was placed in a 45° orientation relative to the muscle fibers associated with the trigger point and held for no more than 24 h (Figure 1B). In the ischemic compression technique, sustained pressure was applied over the affected muscle area for a period of 30 to 90 s (Figure 1C).

For each of the groups, trigger point pain at rest was reliably assessed randomly, by a single blinded evaluator, at four different times: in the pre-treatment period, immediately after, 1–2 weeks after and one month after. Pain intensity was assessed simply, quickly and objectively using a scale (Numerical Pain Rating Scale—NPRS) from 0 to 10, where 0 represents no pain and 10 represents maximum pain, and patients were asked to assign a numerical value. All treatments were carried out by the same operator, who was responsible for project management.

### 2.4 Data collection and processing

Data collected at the specified time intervals were systematically structured and organized in Excel tables, followed by a comprehensive statistical analysis. Descriptive statistics were employed to characterize the sample, using measures such as mean, standard deviation, and 25th and 75th percentiles, reflecting the quantitative nature of the variables. Intragroup comparisons between different time points within each therapeutic strategy were performed using Friedman's test for paired samples, supplemented by Dunn's test. This approach was necessitated by the violation of the normality assumption, determined by the Shapiro-Wilk test, which indicated that the data did not conform to a normal distribution and required the use of nonparametric tests. For comparisons between groups at the different time intervals, a linear mixed-effects model was applied. Statistical analyses were performed with GraphPad Prism 9.0 and MS® Excel® programs, with a significance threshold set at *p* < 0.05.

## 3 Results

**Figure 2:**
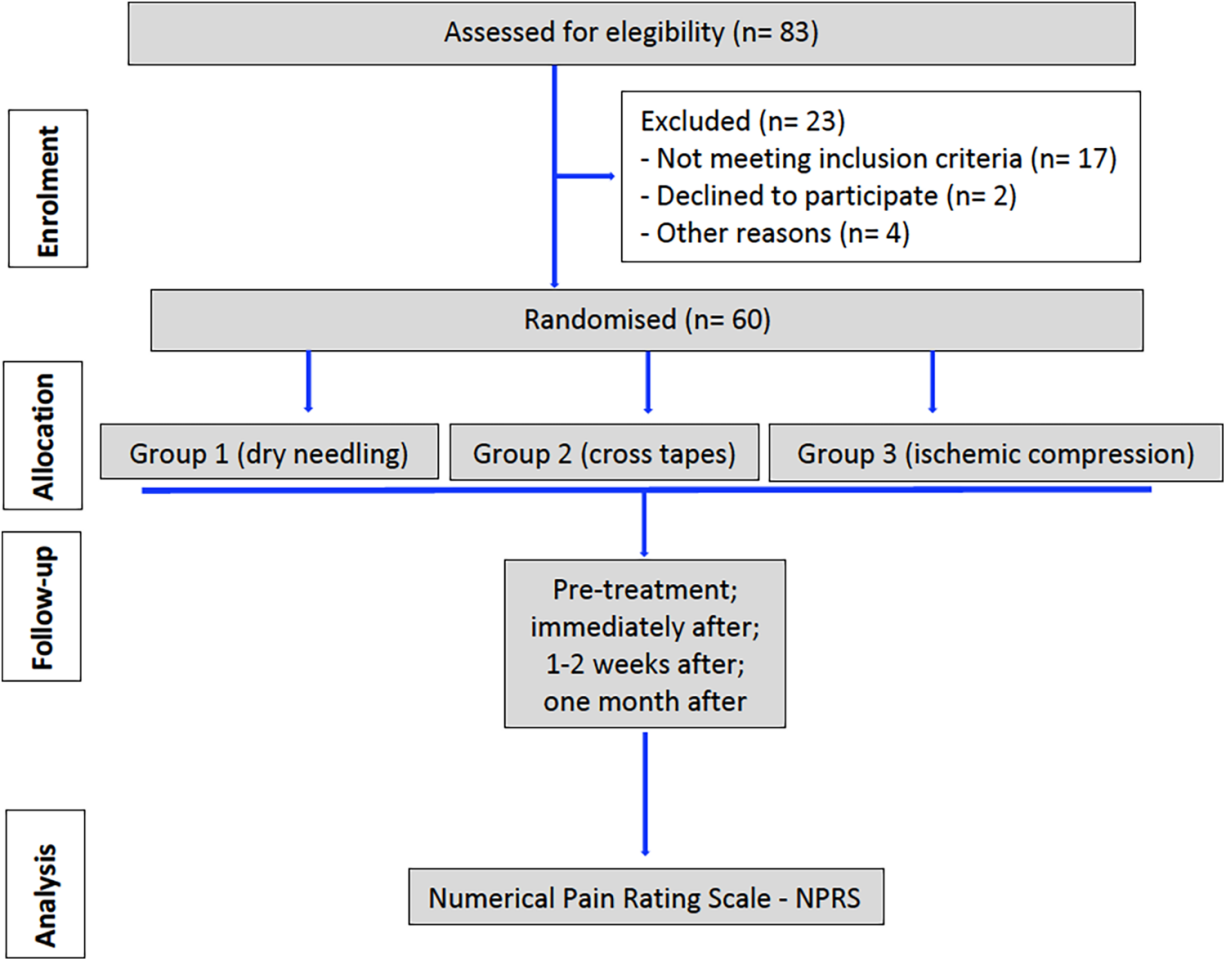
Flow chart of progress through the phases of the study according to the CONSORT statement.

There were no dropouts, so data from 60 subjects were available to evaluate the results. The flow diagram of the study has been described according to CONSORT guidelines and is shown in Figure 2. The discrepancy between the three outcome assessors (BMS, AL-V, NL-V) was <85% [Cohen's kappa coefficient (κ)].

Each group consisted of 20 patients. The predominant gender was female, as a considerable majority of the participants in each group were women (80% of the total sample): the dry needling group consisted of 17 women (85%), with a mean age of 36.8 ± 15.88 years. The ischemic compression group consisted of 14 women (70%), with a mean age of 32.3 ± 7.82 years. Finally, the cross-taping group consisted of 17 women (85%), with a mean age of 39.4 ± 19.9 years (Table 1).

**Table 1:** Baseline characteristics of patients.

Group	Patients (n)	Sex (male/female)	Age (years)
Needling group	20	17 Female 3 Male	36.8 ± 15.88
Ischemic compression	20	14 Female 6 Male	32.3 ± 7.82
Cross-taping	20	17 Female 3 Male	39.4 ± 19.9

### 3.1 Pain intensity

The different therapies were applied to the respective groups only once, and pain intensity was assessed at the aforementioned times (pre-treatment period, immediately after, 1–2 weeks after and one month after). The statistical analysis of pain intensity is summarized in Table 2.

**Table 2:** Average pain intensity values stratified by different evaluation periods of time and treatment type.

Treatment	Before	Immediately after	1–2 weeks	1 month
Dry Needling	5.450 ± 0.2945 [95% CI (4.834; 6.066)]	1.400 ± 0.5150 [95% CI (0.3220; 2.478)]	3.550 ± 0.3589 [95% CI (2.799; 4.301)]	3.400 ± 0.3509 [95% CI (2.665; 4.135)]
Ischemic Compression	4.450 ± 0.2112 [95% CI (4.008; 4.892)]	1.950 ± 0.5154 [95% CI (0.8712; 3.029)]	3.200 ± 0.3044 [95% CI (2.563; 3.837)]	2.900 ± 0.2705 [95% CI (2.334; 3.466)]
Cross-Taping	5.950 ± 0.4500 [95% CI (5.008; 6.892)]	5.900 ± 0.4583 [95% CI (4.941; 6.859)]	4.650 ± 0.5195 [95% CI (3.563; 5.737)]	4.800 ± 0.4845 [95% CI (3.786; 5.814)]

### 3.2 Dry needling treatment

**Figure 3:**
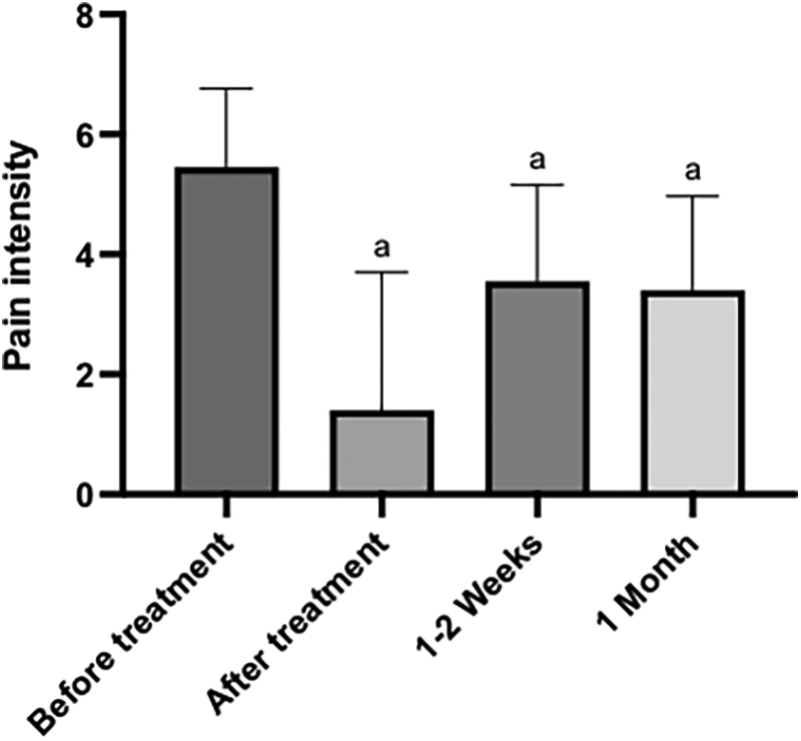
Graph of the variation in pain intensity, in different periods of time, before and after the application of the dry needling technique. The “a” letter indicates groups where differences were statistically significant (*p* < 0.05).

Regarding dry needling, we found a statistically significant decrease in pain intensity comparing the time before and immediately after treatment (*p* < 0.0001); (symbolized by “a”, lower case letter depicted in Figure 3), along with a statistically significant increase in pain intensity comparing the time before and 1–2 weeks after treatment (*p* = 0.0036) and a statistically significant decrease in pain intensity comparing the time before and 1 month after treatment (*p* = 0.0005). However, we did not find statistically significant differences when comparing pain intensity immediately after treatment, with 1–2 weeks after (*p* value = 0.3003), pain intensity immediately after treatment and 1 month after (*p* value = 0.8499) or pain intensity 1–2 weeks after treatment and 1 month after (*p* value > 0.9999).

### 3.3 Ischemic compression treatment

**Figure 4:**
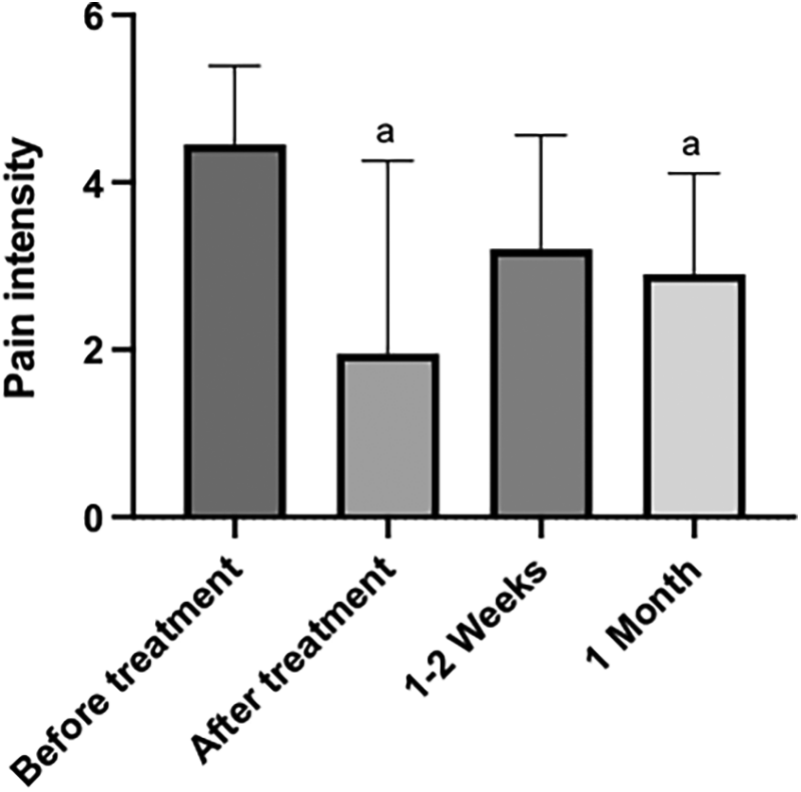
Graph of the variation in pain intensity, in different periods of time, before and after the application of the ischemic compression technique. The “a” letter indicates groups where differences were statistically significant (*p* < 0.05).

With respect to ischemic compression, we observed a statistically significant decrease in pain intensity, comparing the time before and immediately after treatment (*p* = 0.0001). Similarly, we found a statistically significant decrease in pain intensity, comparing the time before and 1 month after treatment (*p* = 0.0023). However, we did not find statistical significance when comparing pain intensity before treatment and 1–2 weeks after (*p* = 0.0723), pain intensity immediately after and 1–2 weeks after (*p* = 0.5185), pain intensity immediately after and 1 month after (*p* > 0.9999), or pain intensity values 1–2 weeks after treatment and 1 month after (*p* > 0.9999) (Figure 4).

### 3.4 Cross-taping treatment

**Figure 5:**
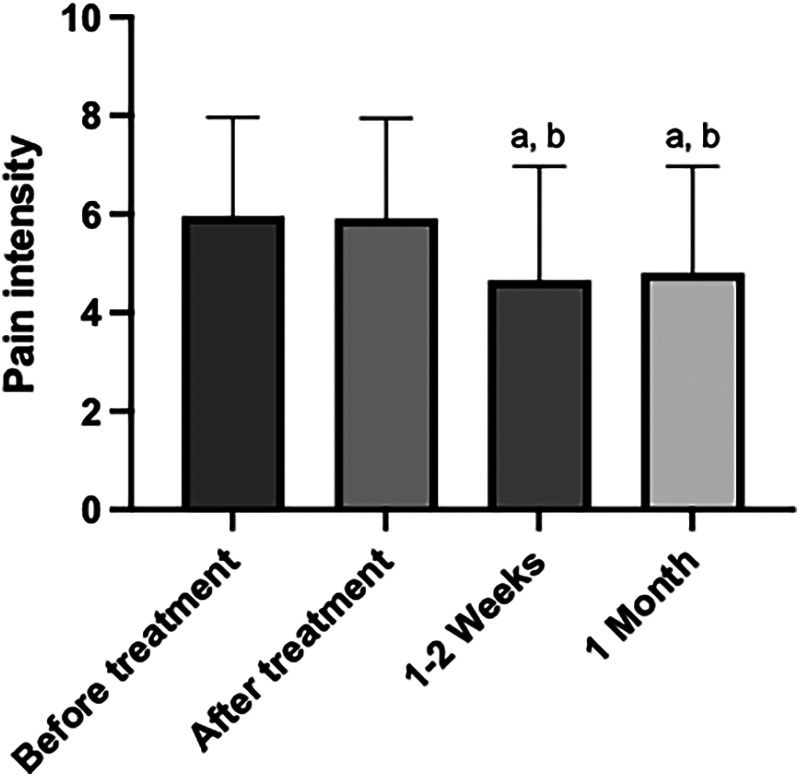
Graph of the variation in pain intensity, in different periods of time, before and after the application of the cross-taping technique. The “a” and “b” letter indicates groups where differences were statistically significant (*p* < 0.05).

We found a statistically significant decrease in pain intensity, comparing the time before and 1–2 weeks after treatment (*p* = 0.0014). We also found statistical significance in the reduction of pain intensity, comparing the time immediately after treatment and 1–2 weeks after (*p* = 0.0029); (symbolized by “b”, lower case letter depicted in Figure 4). Also, a statistically significant increase in pain intensity comparing the time before and 1 month after treatment (*p* = 0.0029) and a statistically significant decrease in pain intensity comparing the time immediately after treatment and 1 month after (*p* value = 0.0057). We found no statistically significant differences when comparing pain intensity at the time before and immediately after treatment (*p* > 0.9999), and pain intensity 1–2 weeks after treatment and 1 month after (*p* value > 0.9999) (Figure 5).

Considering the results evaluated and comparing the three treatments applied, it was observed that immediately after, there was a statistically significant difference in pain intensity between the cross-taping technique and dry needling (*p* < 0.0001) and between the cross-taping technique and ischemic compression (*p* < 0.0001). After 1–2 weeks, there was only a statistically significant difference in pain intensity between the cross-taping technique and ischemic compression (*p* = 0.0337). Finally, after 1 month, there was a statistically significant difference in pain intensity between cross-taping and dry needling (*p* = 0.0422) and between cross-taping and ischemic compression (*p* = 0.0033). In the comparisons not mentioned, the *p* value > 0.05, therefore, were not significant.

**Figure 6:**
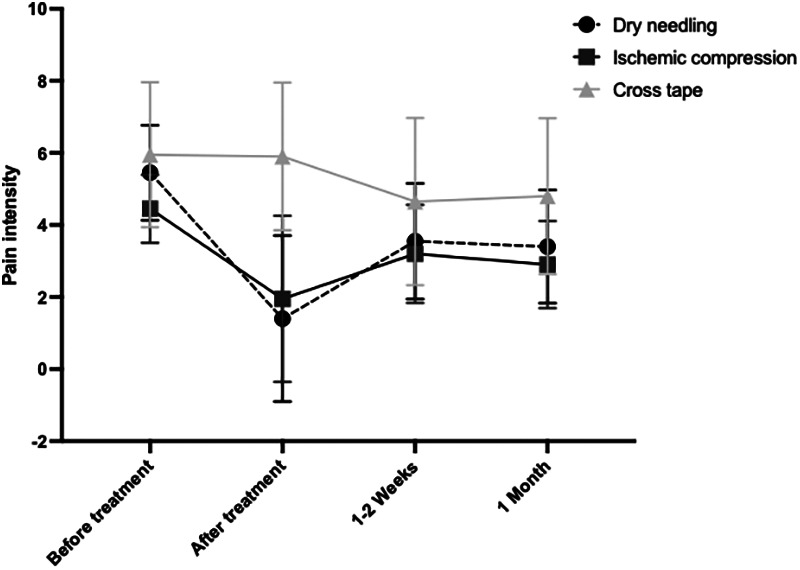
Line graph on the variation of pain intensity in different periods of time, before and after the application of the three techniques described.

According to the results obtained, it was observed that, immediately after applying the therapies, there was a greater decrease in pain intensity in dry needling, followed by ischemic compression and a less pronounced decrease in the cross-taping technique. After 1–2 weeks, there was a noticeable increase in dry needling and ischemic compression, being greater in the former mentioned. In the case of the cross-taping technique, there was a decrease in pain intensity. Finally, after 1 month of application, both dry needling and ischemic compression showed a slight reduction in pain intensity, in contrast to the cross-taping group, which showed an increase in pain intensity (Figure 6).

## 4 Discussion

The most prevalent muscular disorder in the population corresponds to the taut band, from which the concept of a myofascial trigger point may emerge (44). Therefore, the need for this study is mainly justified by the fact that hyperirritable nodules are very frequent in the general population and, consequently, can cause significant disturbances in people's quality of life (45).

In addition, its complex and demanding treatment requires a careful and effective approach by dental professionals (8). Rodriguez-Mansilla et al. (20) in a meta-analysis on a sample of 10 studies concluded that dry needling (DN would be convenient this acronym) is more beneficial in reducing pain intensity immediately after the intervention compared to placebo and control groups, despite not being significantly different from placebo in pain reduction after 3–4 weeks. These results are in agreement with our investigation, which found a statistically significant decrease in pain intensity (*p* < 0.0001) immediately after the intervention. However, at follow-up assessments (1–2 weeks and 1 month later), pain intensity increased, although it remained below baseline levels (before applying the treatment technique). These same authors in their meta-analysis suggest that other therapeutic strategies such as active stretching exercises, ultrasound therapy, injections with analgesics, lidocaine or corticosteroids, are more effective in reducing pain intensity immediately after treatment and during the following 3–4 weeks, compared to dry needling, which, although it produces a significant improvement immediately after the procedure, is not long lasting. Therefore, although clinical practice shows that this technique is increasingly used with beneficial effects in the treatment of trigger points, the available scientific evidence does not provide sufficiently consistent and solid results on its medium and long-term efficacy. Another large meta-analysis by Vier et al. (35), comparing dry needling with sham therapy in terms of pain intensity and maximum pain-free mouth opening, showed no statistically significant differences in the short term. However, dry needling showed a better effect on pain threshold to pressure, indicating greater short-term pain tolerance compared with sham therapy. The meta-analysis also showed that laser therapy is more effective than dry needling, probably due to benefits on microcirculation, which would improve oxygen delivery to hypoxic cells and aid in the removal of cellular metabolic waste. Although laser therapy is preferable for needle-phobic patients and for healthcare professionals inexperienced with dry needling, further clinical studies are needed to conclusively demonstrate its superiority over dry needling. A meta-analysis evaluating the efficacy of dry needling for myofascial trigger points reported that it reduced pain intensity immediately and in the short term compared with placebo and other needling techniques, such as acupuncture and ischemic compression (23). In our investigation we found that dry needling produced better results in reducing pain intensity compared to ischemic compression. However, 1–2 weeks later, pain levels increased in both groups, with a more significant increase in the dry needling group. Consistent with Viel et al., although dry needling initially increased the pain threshold to pressure, this effect was not sustained in the short term compared to the other groups, except for ischemic compression, which showed similar results. These findings align with recent theories suggesting a common neurophysiological mechanism between manual therapy and needling approaches, which helps to reduce the perception or response to pain (46, 47). A recent meta-analysis conducted by Lu W et al. (38) that assessed pain intensity on the Visual Analogue Scale (VAS) and the Numerical Pain Rating Scale (NPRS) concluded that ischemic compression was not effective in reducing pain intensity, as there were no significant differences between the therapy and control groups, however, our research found that ischemic compression effectively reduced pain immediately after application, although pain increased after 1–2 weeks, with a slight decrease observed after one month. We also found an improvement to pain tolerance, observed in the increase of pain threshold to pressure. The different results may be due to the fact that ischemic compression does not inhibit central nervous system sensitization, which may persist after myofascial release, indicating that the reported pain may be due to central sensitization or hyperexcitability. García-de la-Banda-García et al. (10) observed that both dry needling and ischemic compression were effective in reducing pain intensity and related disability, with no significant differences between the two at the end of treatment, which they estimated at three sessions, with four days of interval between them. However, this effect was not observed after the first session, possibly due to the adverse effects of dry needling, such as residual pain. They also observed that both techniques effectively reduced pain sensitivity, with no significant differences. Although their protocol differed from ours, we found an immediate reduction in pain after dry needling, followed by ischemic compression, and in both treatments, we observed an increase in pain intensity after 1–2 weeks, with a slight decrease after one month. The literature reviewed revealed significant information gaps regarding the cross-taping technique, as the evidence provided does not support conclusive statements about its efficacy on the masseter muscle. Lee (41) in an editorial published in 2021, only describes the format and method of application of cross-taping, along with some precautions, serving more as a protocol with guidelines, rather than evidence of its efficacy. Lietz-Kijak et al. (22), in a prospective study on 60 adult patients with TMDs, evaluated the efficacy of kinesio-taping, a method similar to cross-taping, which consists in the application of specific tapes on the skin. With this method they mainly aimed to normalize muscle tension, improve the function of weakened muscles and improve microcirculation at the site of application, demonstrating the efficacy of kinesio-taping on pain intensity; however, in our study, we observed that ischemic compression produced a significantly greater reduction in pain intensity, immediately after treatment, compared to cross-taping (*p* < 0.001). We also observed similar effects 1–2 weeks and 1 month later (*p* = 0.0337 and *p* = 0.0033, respectively).

It is important to note that most applications of cross-taping in the literature focus on other muscles, such as the sternocleidomastoid, upper trapezius, anterolateral aspect of the thigh, and the intrinsic and extrinsic muscles of the shoulder, neck and lower back. This study is one of the first to apply cross-taping to the masseter muscle in cases of TMD; therefore, in the absence of further studies, it is difficult to obtain conclusive results evaluating the efficacy of the method in these pathologies. The lack of information on this technique complicates its wider clinical applicability, as the literature focuses mainly on clinical protocols without addressing its efficacy or possible outcomes.

Nevertheless, despite the limitations, the findings of our study are clinically relevant, as they demonstrate that the therapeutic strategies used are effective in the short term for patients. This is of great importance when deciding between invasive treatments, such as dry needling, and noninvasive options, such as ischemic compression and cross-taping, depending on the patient's preferences and the practitioner's experience. Future research should include larger patient groups and longer follow-up periods to assess medium- and long-term outcomes. It is also noteworthy that this study did not measure pain-free maximum mouth opening, lateral movements, or pressure pain thresholds, highlighting areas for future research. Finally, the multidisciplinary nature of TMDs and related trigger points may require a combination of treatment methods, which would result in greater pain relief and thus improved quality of life. This is why synergy between different therapeutic options requires extensive research.

Our study found that dry needling and ischemic compression were more effective than cross-taping for immediate reduction of orofacial myofascial pain. Dry needling showed consistent results, while cross-taping resulted in increased pain after one month. Further research is needed not only to confirm these findings and evaluate their medium- and long-term efficacy, but also to evaluate other parameters such as maximum pain-free mouth opening, or pressure pain thresholds.

## Data Availability

The original contributions presented in the study are included in the article/Supplementary Material, further inquiries can be directed to the corresponding author.
